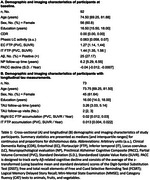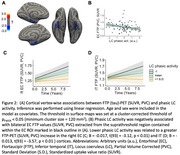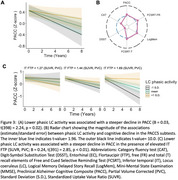# Lower resting‐state phasic locus coeruleus activity is associated with cortical tau accumulation and cognitive decline in preclinical Alzheimer’s disease

**DOI:** 10.1002/alz70862_110117

**Published:** 2025-12-23

**Authors:** Prokopis C. Prokopiou, Aaron P. Schultz, Kathryn V Papp, David H Salat, Dorene M. Rentz, Reisa A. Sperling, Keith A. Johnson, Heidi I.L. Jacobs

**Affiliations:** ^1^ Athinoula A. Martinos Center for Biomedical Imaging, Department of Radiology, Massachusetts General Hospital, Harvard Medical School, Charlestown, MA USA; ^2^ Center for Alzheimer’s Research and Treatment, Department of Neurology, Brigham and Women’s Hospital, Harvard Medical School, Boston, MA USA; ^3^ Center for Alzheimer’s Research and Treatment, Brigham and Women’s Hospital, Harvard Medical School, Boston, MA USA; ^4^ Gordon Center for Medical Imaging, Massachusetts General Hospital, Harvard Medical School, Boston, MA USA; ^5^ Athinoula A. Martinos Center for Biomedical Imaging, Massachusetts General Hospital, Harvard Medical School, Boston, MA USA

## Abstract

**Background:**

The brainstem noradrenergic locus coeruleus (LC) is among the earliest regions to accumulate Alzheimer’s disease (AD)‐related hyperphosphorylated tau. Lower novelty‐related LC activity in older cognitively healthy individuals has been related to greater entorhinal (EC) tau deposition and steeper memory decline. Furthermore, rat models accumulating LC pretangle human tau receiving novelty‐like, phasic optogenetic activation of the LC exhibited resilience against tau’s downstream effects on cognition. Using novel modeling approaches to identify phasic‐like activity from BOLD data, we associated in‐vivo phasic‐like, spontaneous LC activity with cortical tau accumulation and cognitive decline in asymptomatic older adults.

**Method:**

Ninety‐two participants (56 Female, mean age at baseline=75±9.0 years; Table 1) from the Harvard Aging Brain Study underwent FTP(tau)‐PET and 3T resting‐state BOLD‐fMRI imaging (TR/TE=800/37ms, voxel=2mm^3^) at baseline and longitudinal cognitive testing (mean follow‐up=5.37±1.88 years). Seventy‐three participants also had longitudinal FTP‐PET data (mean follow‐up=4.26±1.78 years). Phasic‐like LC activity events were defined at BOLD‐fMRI signal peaks, maximizing activity integration within the EC, using an in‐house‐developed minimum entropy‐based event‐detection algorithm. The proportion of LC activity explained by the detected events was quantified using the R^2^‐coefficient. Vertex‐wise robust regressions associated LC activity with cortical tau deposition, adjusted for age and sex. Mixed‐effects models associated LC activity and EC tau accumulation, as well as LC activity and cognitive decline (PACC5, Table 1), adjusted for age, sex, and education (for cognition models).

**Result:**

Lower phasic LC activity was associated with greater baseline EC tau deposition (Figure 2A‐B) and faster medial temporal lobe tau accumulation (Figure 2C‐D). Lower phasic LC activity was associated with steeper cognitive decline, with larger effect sizes for the memory‐related subtests of PACC5 (Figure 3A‐B). Furthermore, the effect of LC phasic activity on cognitive decline was more pronounced at elevated levels of inferior‐temporal tau (Figure 3C).

**Conclusion:**

In line with previous animal and in‐vivo imaging studies, our results suggest that higher phasic LC activity may be protective against the accumulation of tau in early AD‐related cortical regions and its adverse effects on cognition. The findings in this work could inform the design of early targeted interventions to support optimal LC phasic activity to delay cognitive decline.